# Impact of Pyrethroid Resistance on the Intrinsic Insecticidal Activities of Geraniol Against the Yellow Fever Mosquito, *Aedes aegypti*

**DOI:** 10.3390/insects17040385

**Published:** 2026-04-02

**Authors:** Paola N. Feliciano, Peter M. Piermarini

**Affiliations:** Department of Entomology, College of Food, Agricultural and Environmental Sciences, The Ohio State University, Wooster, OH 44691, USA; felicianocamacho.1@buckeyemail.osu.edu

**Keywords:** mosquito, geraniol, larvicide, adulticide, repellent

## Abstract

The evolution of resistance in mosquitoes to conventional insecticides such as pyrethroids presents a challenge to vector control. Thus, alternative active ingredients for insecticides to manage pyrethroid-resistant populations of mosquitoes are needed. The goal of this study was to evaluate the toxic and repellent efficacy of geraniol, a plant secondary metabolite, as a potential alternative for controlling pyrethroid-resistant *Aedes aegypti*. We found that addition of geraniol to rearing water of 1st instar larvae caused concentration-dependent mortality within 24 h in both strains. The resistance ratio of geraniol (2.8) was modest compared to that of cypermethrin (435.3). Topical application of geraniol to adult female mosquitoes caused dose-dependent mortality in both strains within 24 h. The resistance ratio of geraniol (1.1) was minimal compared to that for cypermethrin (457). In spatial repellency assays, geraniol repelled adult females from both strains in a dose-dependent manner. The repellency resistance ratio of geraniol (2.6) was modest compared to that for pyrethrum extract (>132). Our findings suggest that geraniol has potential use as a toxicant and repellent for controlling pyrethroid-resistant populations of *Ae. aegypti*.

## Simple Summary

The present study compared the toxic and repellent effects of geraniol on *Aedes aegypti* mosquitoes from representative pyrethroid-susceptible and pyrethroid-resistant strains. Although the pyrethroid-resistant strain showed modest resistance to geraniol as a toxin and repellent, the degree of resistance was far lower than that for a pyrethroid or pyrethrum extract. Our results suggest that geraniol has potential use for controlling pyrethroid-resistant populations of *Ae. aegypti*.

## 1. Introduction

Mosquito-borne arboviruses such as dengue, Zika, and chikungunya represent significant global health threats, collectively infecting hundreds of millions of people and causing tens of thousands of deaths annually [1,2,3]. *Aedes aegypti*, the yellow fever mosquito, is one of the most important vectors for transmission of these viruses to humans. Control strategies for *Ae. aegypti* have relied on chemical approaches that use insecticides with limited modes of action, which has led to insecticide resistance in mosquito populations and challenges to the control of mosquito-borne arboviral diseases [4,5,6]. Insecticide resistance in mosquitoes can arise through multiple mechanisms, including target-site (e.g., knockdown resistance, *kdr*, mutations in voltage-gated sodium channels), metabolic (e.g., overexpression of detoxification enzymes, such as cytochrome P450 monooxygenases), and cuticular (e.g., thicker cuticle that reduces penetration of insecticides) [7,8].

Pyrethroids, such as deltamethrin, alpha-cypermethrin, and lambda-cyhalothrin, are commonly used for mosquito control [9], which has resulted in widespread pyrethroid resistance in *Ae. aegypti* and other mosquito species. In addition, overuse of pyrethroids has raised concerns about threats to non-target species and environmental health. Pyrethroids act on neural signaling proteins, the voltage-gated sodium channels, which are conserved across other arthropods and vertebrates, including humans [4,10,11,12]. As a result, there is a need to develop new environmentally friendly chemical tools for vector control.

Plant essential oils and their constituents represent a promising source of bioactive compounds for developing new pesticides. Essential oils are rich in secondary metabolites, which often play crucial roles in the chemical defenses of plants [13,14,15,16,17]. Some secondary metabolites exhibit insecticidal activity, making them potential active ingredients for developing biopesticides. Furthermore, some secondary metabolites are considered safer for human health, less harmful to non-target organisms, and more environmentally sustainable than conventional synthetic insecticides [13,15].

Geraniol is a common, abundant secondary metabolite found in essential oils of several plants, including geranium (*Pelagornium* sp.), citronella (*Cymbopogon* sp.), and rose (*Rosa* sp.) [18,19,20,21]. This compound is a monoterpene alcohol with a rose-like scent that is listed by the U.S. Environmental Protection Agency (EPA) as a minimum risk pesticide [22,23,24,25]. Essential oils rich in geraniol have larvicidal and ovicidal effects against several species of mosquitoes, including *Culex pipiens*, *Cx. quinquefasciatus*, *Ae. albopictus*, and *Ae. aegypti* [26,27,28,29]. Consistent with these studies, geraniol has larvicidal activity against multiple mosquito species, including *Cx. pipiens*, *Cx. quinquefasciatus*, *Anopheles stephensi*, *Ae. albopictus*, and *Ae. aegypti* [22,26,27,30,31]. Moreover, geraniol and/or essential oils enriched with geraniol have adulticidal activity [29,32]. However, a limitation of these prior studies is that they were conducted primarily on insecticide-susceptible mosquito strains. Thus, the toxic efficacy of geraniol against insecticide-resistant mosquitoes is underexplored.

Prior studies have also investigated geraniol’s potential use as a mosquito repellent, which has yielded mixed results. For example, some studies have found that geraniol is a relatively strong repellent, outperforming other natural repellents such as citronella and linalool against susceptible strains of *Ae. aegypti* [15,25]. However, another study found geraniol to be an ineffective repellent against *Ae. aegypti* [33]. Intriguingly, in *An. gambiae*, geraniol showed similar repellent efficacy against both pyrethroid-susceptible and pyrethroid-resistant strains, but weaker repellency against an organophosphate-resistant strain [4]. Thus, our understanding of the potential use of geraniol as a repellent is limited and requires further investigation, especially against insecticide-resistant mosquitoes. 

The goal of this study was to address the above research gaps by comparing the larvicidal, adulticidal, and repellent effects of geraniol between representative pyrethroid-susceptible and pyrethroid-resistant strains of *Ae. aegypti*. We hypothesized that pyrethroid-resistant *Ae. aegypti* would exhibit toxic resistance to geraniol, because a previous computational study [34] predicted that geraniol potentially interacts with biochemical mechanisms that contribute to pyrethroid resistance, such as voltage-gated sodium channels involved with knockdown resistance and detoxification enzymes (glutathione *S*-transferase & esterase) involved in metabolic resistance. On the other hand, we hypothesized that pyrethroid-resistant *Ae. aegypti* would show limited repellency resistance to geraniol, because a previous study in *An. gambiae* [4] found that geraniol was equally effective as a repellent against pyrethroid-susceptible and pyrethroid-resistant strains.

## 2. Materials and Methods

### 2.1. Ae. aegypti Colonies and Strains

Two different strains of *Ae. aegypti* mosquitoes were used in the present study: Liverpool (LVP-IB12, MRA-735, obtained by David W. Becnel) and Puerto Rico (PR-NR-48830, obtained by G.G. Clark & J.J. Becnel). The Puerto Rico strain is highly resistant to pyrethroids via both target site (*kdr*) and metabolic resistance [35,36,37]. From here, we refer to the Liverpool strain as pyrethroid-susceptible (PS) and the Puerto Rico strain as pyrethroid-resistant (PR). To rear mosquitoes, larvae from both strains were fed with 1 tablet per day of commercial fish food (Tropical Tablets, Tetramin, Blacksburg, VA, USA). Adult mosquitoes were maintained on 10% sucrose; for egg production, adult females were fed defibrinated rabbit blood (Hemostat Laboratories, Dixon, CA, USA) via a membrane feeder (Hemotek Ltd., Blackburn, UK). All mosquito colonies were maintained in environmentally-controlled rearing chambers set at the following conditions: 28 °C, 80% relative humidity, and 12:12 light: dark cycle.

### 2.2. Chemicals

Geraniol (≥99%, (2E)-3,7-dimethylocta-2,6-dien-1-ol) was purchased from Thermo Fisher Scientific (Waltham, MA, USA). Cypermethrin (>96%) was obtained from Thermo Fisher Scientific (Acros Organics, Geel, Belgium). Transfluthrin (≥99%) was obtained from Thermo Fisher Scientific (Chicago, IL, USA). Pyrethrum extract (≥50% sum of pyrethrins) was purchased from Sigma-Aldrich (St. Louis, MO, USA).

### 2.3. Larvicidal Bioassay

**Figure 1:**
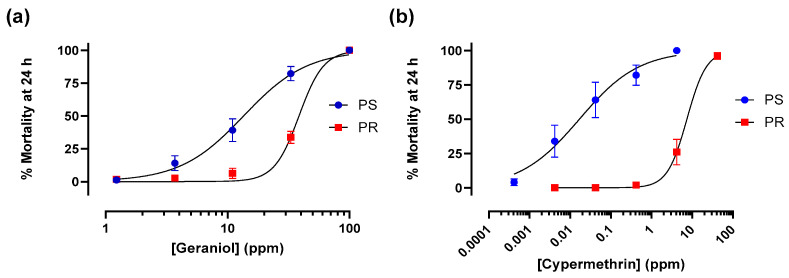
Concentration-dependent mortality (24 h) of (**a**) geraniol and (**b**) cypermethrin against 1st instar larvae of pyrethroid-susceptible (PS, blue) and pyrethroid-resistant (PR, red) *Ae. aegypti*. For geraniol, values are means ± SEM based on 32 replicates per concentration for the PS strain and 20 replicates per concentration for the PR strain. For cypermethrin, values are means ± SEM based on 20 replicates per concentration for both the PS and PR strains. Each replicate consisted of one well of a 24-well plate with 5 larvae. No mortality was observed in solvent controls.

Larvicidal activities of geraniol and cypermethrin were tested against 1st-instar larvae (mixed sex, 24 h post-hatching) of PS and PR strains using an established bioassay [14,38]. Cypermethrin was used as a positive control/reference compound for confirming toxic resistance of larvae of the PR strain [38,39]. In brief, 5 larvae were added to wells of 24-well Falcon Multiwell plates (Becton Dickinson Labware, Franklin Lakes, NJ, USA) containing 985 µL of distilled water and 5 µL of a food suspension. The food suspension consisted of 13 mg of finely ground fish food flakes (Tetramin, Blacksburg, VA, USA) suspended in 1 mL of deionized water. After adding larvae, solvent control wells received 10 µL of 100% acetone, whereas treatment wells received 10 µL of geraniol or cypermethrin dissolved in 100% acetone at various concentrations (freshly prepared on the day of testing). The final concentrations of geraniol in the wells were 1.2, 3.7, 11, 33, or 100 ppm; the final concentrations of cypermethrin in the wells were 0.0004, 0.004, 0.04, 0.4, 4.0, or 40 ppm. The plates were held in the rearing chambers and larval mortality was assessed 24 h post-treatment. Larvae were classified as dead if they failed to respond after gently probing with a pipette tip. For each experimental trial, a chemical concentration (or control) was tested in quadruplicate on a 24-well plate. A total of at least 5 independent trials (i.e., 20 wells total) were performed for each concentration and strain. See legend of Figure 1 for details.

### 2.4. Topical Adulticidal Bioassay

Topical adulticidal activities of geraniol and cypermethrin were tested against adult females of PS and PR strains using an established bioassay [40,41]. Cypermethrin was used as a positive control/reference compound for confirming toxic resistance of adult females of the PR strain [39]. Ten mosquitoes (3–10 days post-eclosion) were briefly immobilized on ice and 500 nL of 100% acetone (solvent control) or a treatment (geraniol or cypermethrin dissolved at various concentrations in 100% acetone) were applied to the thorax of each mosquito using a Hamilton repeating dispenser (Hamilton Company, Reno, NV, USA). The doses of geraniol were 0.20, 0.55, 1.85, 5.50, and 16.5 µg/mosquito; the doses of cypermethrin were 0.00019, 0.0019, 0.019, 0.19, 1.9, and 19.2 ng/mosquito.

**Figure 2:**
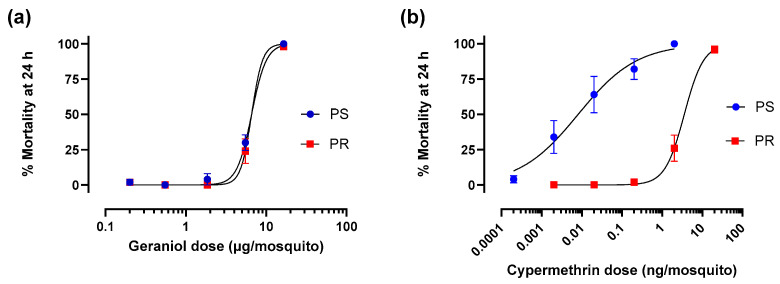
Dose-dependent mortality (24 h) of (**a**) geraniol and (**b**) cypermethrin against adult females of pyrethroid-susceptible (PS, blue) and pyrethroid-resistant (PR, red) *Ae. aegypti*. Values are means ± SEM based on 5 replicates per dose for both geraniol and cypermethrin for both strains. Each replicate consisted of one cage of 10 mosquitoes. No mortality was observed in solvent controls.

After treating the mosquitoes, they were transferred to small cages (32 oz. containers) and provided with access to 10% sucrose. The cages were held in the rearing chamber and toxicity was evaluated based on the percentage of mosquitoes that were dead 24 h post-treatment. For each experimental trial, a chemical dose (or control) was tested on a cage of 10 mosquitoes. A total of at least 5 independent trials were performed for each dose and strain. See legend of Figure 2 for details.

### 2.5. Mosquito Airborne Repellency Test (MART Assay)

Spatial or airborne repellency of geraniol and pyrethrum extract was tested using a previously described method [42], with the minor modification that 12 mosquitoes were used per replicate instead of 16. Transfluthrin was initially used as a positive control/reference compound for confirming repellency resistance of adult females of the PR strain [43]. However, in preliminary experiments, we did not detect repellency of this compound in either the PS or PR strain, which may have been attributed to its vapor toxicity, resulting in immobile or knocked down mosquitoes. Thus, we switched to pyrethrum extract as a positive control/reference compound for repellency [43]. Groups of twelve adult females were transferred into glass cylinders (length 12.5 cm, outer diameter 2.5 cm; TriKinetics Inc., Walthman, MA, USA) with mesh netting secured on both ends with rubber bands (the netting was temporarily removed from one end to introduce the mosquitoes). Before adding the mosquitoes, a line was drawn on each tube at the half-way point of its length. Mosquitoes were acclimated in the tubes for 30 min in a rearing chamber (28 °C, 80% relative humidity) before starting experiments.

While mosquitoes were acclimating to the tubes, fifty microliters of 100% acetone (solvent control) or a treatment (geraniol or pyrethrum extract dissolved in 100% acetone) were applied to round filter papers (diameter 2.5 cm; Cytiva, Fisher Scientific, Waltham, MA, USA). Geraniol was applied at 0.5, 1.0, 3.8, 12.0, and 38.0 µg/cm^2^ for experiments on PS mosquitoes, and 3.8, 12.0, 38.0, 112.0, and 336.0 µg/cm^2^ for experiments on PR mosquitoes. Pyrethrum extract was applied at 1.1, 3.3, 12.0, 33.0, and 127.3 µg/cm^2^ for experiments on PS mosquitoes, and 101.0, 254.6, 336.0, 509.2, 1018.3, and 2036.7 µg/cm^2^ for experiments on PR mosquitoes. The filter papers were placed in plastic caps from 50 mL polypropylene centrifuge tubes (VWR, Radnor, PA, USA) under a fume hood for 10 min to allow acetone to evaporate.

To initiate an experiment, one end of a glass tube containing mosquitoes was fitted with a cap containing the treatment filter paper, whereas the other end was fitted with a cap containing the solvent control filter paper. In addition, to confirm that mosquitoes showed no preference towards either end of the tube, double-solvent controls were performed in which both ends were fitted with caps containing filter papers treated with 100% acetone. Fifteen minutes after fitting the caps, the numbers of mosquitoes on the treatment and control halves of the tubes were counted and the repellency of the treatment was calculated using Formula (1). Any mosquitoes that were knocked down (i.e., not upright, suggesting intoxication or poor health) were excluded. In the double-solvent controls and pyrethrum extract experiments, no knock down of mosquitoes was observed. In geraniol experiments, a few individuals of the PR strain were knocked down at the two highest doses. That is, in the 112.0 µg/cm^2^ treatment, knocked-down mosquitoes were observed in 2 of 6 replicates (1 or 2 individuals). In the 336.0 µg/cm^2^ treatment, a few knocked-down mosquitoes were observed in each replicate: 2 individuals in 5 of the 6 replicates and 1 individual in 1 of the 6 replicates. The double-solvent controls for each experiment showed minimal repellency for both the PS and PR mosquitoes (Figure S1), indicating no preference of mosquitoes towards either end of the tubes in the absence of geraniol or pyrethrum extract.

(1) $$\mathrm{Percent}\mathrm{repellency}=\left(\frac{\mathrm{number}\;\mathrm{of}\;\mathrm{mosquitoes}\;\mathrm{on}\;\mathrm{control}\;\mathrm{side}-\mathrm{number}\;\mathrm{of}\;\mathrm{mosquitoes}\;\mathrm{on}\;\mathrm{treatment}\;\mathrm{side}}{\mathrm{total}\;\mathrm{number}\;\mathrm{of}\;\mathrm{mosquitoes}}\right)\times 100$$

### 2.6. Statistical Analysis

All data were analyzed using Prism 10 (GraphPad Software). For toxicity assays, the median lethal concentrations (LC_50_) for larvae or doses (LD_50_) for adults were determined by plotting percent mortalities against log transformations of the treatment tested and fitting a non-linear regression (log(agonist) vs. normalized response function). For repellency assays, the median effective doses (ED_50_) were calculated similarly. Statistical comparisons of the LC_50_, LD_50_, or ED_50_ values between the PS and PR strains were compared through extra sum-of-squares F-tests (α = 0.05).

## 3. Results

### 3.1. Toxicity of Geraniol and Cypermethrin to PS and PR Larvae

Addition of geraniol to the rearing water of first-instar *Ae. aegypti* larvae resulted in concentration-dependent mortality within 24 h in both the PS and PR strains (Figure 1a). The LC_50_ of geraniol for the PS strain (13.6 ppm, 95% CI: 11.0–16.6 ppm) was lower (*p* < 0.0001) than that of the PR strain (38.6 ppm, 95% Cl: 33.7–44.1 ppm), indicating a larvicidal resistance ratio of 2.8. To provide context for the larvicidal resistance ratio of geraniol, similar experiments were performed using cypermethrin. Previously, we found the resistance ratio of larvae from this same PR strain ranged from 30 to 131 for cypermethrin [38,39]. As shown in Figure 1b, the 24 h LC_50_ of cypermethrin for the PS strain in the present study was 0.02 ppm (95% CI: 0.01–0.04 ppm), whereas that for the PR strain was dramatically higher (*p* < 0.0001) at 7.40 ppm (95% CI: 1.01–10.16 ppm), indicating a resistance ratio of 435.3.

### 3.2. Toxicity of Geraniol and Cypermethrin to PS and PR Adult Females

Topical application of geraniol to adult female *Ae. aegypti* resulted in dose-dependent mortality within 24 h in both the PS and PR strains (Figure 2a). The LD_50_ for the PS strain (6.5 µg/mosquito, 95% CI: 5.1–8.3 µg/mosquito) was similar (*p* = 0.6) to that for the PR strain (7.1 µg/mosquito, 95% CI: 5.7–8.8 µg/mosquito), indicating an adulticidal resistance ratio of 1.1. To provide context for the adulticidal resistance ratio of geraniol, similar experiments were performed using cypermethrin. Previously, we found the resistance ratio of adult females from this same PR strain of *Ae. aegypti* to cypermethrin was 84 [39]. As shown in Figure 2b, the 24 h LD_50_ of cypermethrin for the PS strain in the present study was 0.0078 ng/mosquito (95% CI: 0.0034–0.0176 ng/mosquito), whereas that for the PR strain was dramatically higher (*p* < 0.0001) at 3.55 ng/mosquito (95% CI: 2.42–5.10 ng/mosquito), indicating a resistance ratio of 457.

### 3.3. Spatial Repellency of Geraniol and Pyrethrum Extract to PS and PR Adult Females

**Figure 3:**
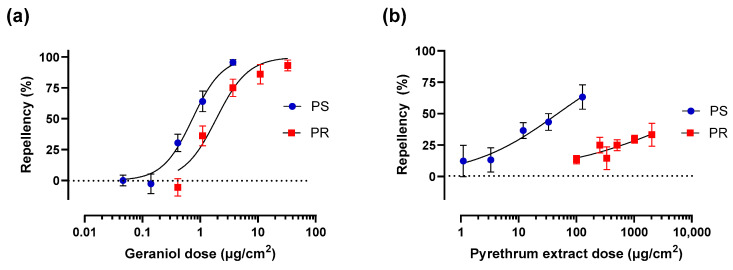
Dose-repellency (15 min) curves of (**a**) geraniol and (**b**) pyrethrum extract against adult females of pyrethroid-susceptible (PS, blue) and pyrethroid-resistant (PR, red) *Ae. aegypti.* For geraniol, values represent mean ± SEM based on 6–12 replicates per dose for PS and 6 replicates per dose for PR. For pyrethrum extract, values represent mean ± SEM based on 5 replicates per dose for PS and 5–8 replicates per dose for PR. Each replicate consisted of one tube of 12 mosquitoes. Figure S1 shows the respective double-solvent controls for the geraniol and pyrethrum extract experiments.

Adult female *Ae. aegypti* from both PS and PR strains were spatially repelled by geraniol in a dose-dependent manner (Figure 3a). The ED_50_ for the PS strain (0.74 µg/cm^2^, 95% Cl: 0.58–0.95 µg/cm^2^) was lower (*p* < 0.0001) than that for the PR strain (1.90 µg/cm^2^, 95% CI: 1.38–2.63 µg/cm^2^), indicating a repellency resistance ratio of 2.6.

To provide context for the repellency resistance ratio of geraniol, similar experiments were performed using pyrethrum extract. Previously, Yang et al. [43] found the repellency resistance ratio of adult females from this same PR strain of *Ae. aegypti* to pyrethrum extract was 7.9 relative to the pyrethroid-susceptible Orlando strain of *Ae. aegypti*. As shown in Figure 3b, the ED_50_ of pyrethrum extract for the PS strain was 47.36 µg/cm^2^ (95% CI: 23.82–140.7 µg/cm^2^). In contrast, pyrethrum extract showed weak dose-dependent spatial repellency against the PR strain that did not exceed 30%, indicating an ED_50_ that is beyond detectable limits. Based on the highest dose used in the present study (2036.7 µg/cm^2^) we can assume the repellency resistance ratio of pyrethrum extract is at least 132. Attempts to use higher doses of pyrethrum extract to drive higher repellency responses for both the PS and PR strains were unsuccessful due to vapor toxicity of the extract.

## 4. Discussion

The present study found that geraniol elicited concentration-dependent larvicidal activity against both PS and PR *Ae. aegypti*. The results for PS larvae were consistent with previous studies that found concentration-dependent larvicidal activity of geraniol against pyrethroid-susceptible strains of *Cx. pipiens*, *Cx. quinquefasciatus*, *An. stephensi*, *Ae. albopictus*, and *Ae. aegypti* [23,26,30,31]. Notably, larvae of the PR strain possessed modest cross-resistance (2.8 resistance ratio) to the toxicity of geraniol, but this was much lower than that for cypermethrin, a pyrethroid (435.3 resistance ratio). We also found that geraniol elicited dose-dependent adulticidal activity against both PS and PR *Ae. aegypti*. The results for PS adults were consistent with previous studies that found dose-dependent adulticidal activity in pyrethroid-susceptible strains of *Cx. pipiens*, *Ae. aegypti*, and *An. quadrimaculatus* [29,32]. In contrast to the larvicidal results, we found that adult females of the PR strain did not exhibit detectable cross-resistance to geraniol (1.1 resistance ratio) while they maintained strong resistance to cypermethrin (457 resistance ratio).

The PR strain of *Ae. aegypti* used in the present study (Puerto Rico) possesses target site resistance via *kdr* mutations in voltage-gated sodium channels and metabolic resistance likely via elevated expression of numerous CYP450 mRNAs and at least one GST mRNA [44]. Approximately 50% of the total resistance in the PR strain is attributed to enhanced CYP450 activity [44]. Cuticular resistance has been documented in other pyrethroid-resistant strains of *Ae. aegypti* [45,46], but it is unknown whether it is present in the Puerto Rico strain used in the present study. Thus, the relatively low toxic resistance ratios for geraniol we observed in the PR strain (compared to those for cypermethrin) suggest that geraniol bypasses most of the resistance mechanisms in larvae and nearly all of the resistance mechanisms in adult females. This suggests that geraniol has strong potential as a toxin to control pyrethroid-resistant populations of *Ae. aegypti*.

To understand why larvae, but not adult females, of the PR strain exhibited modest cross-resistance to geraniol will require further investigation into geraniol’s mode of toxic action and how mosquitoes detoxify the compound. To date, biochemical targets of geraniol in mosquitoes remain unclear, but geraniol is known to inhibit acetylcholinesterase in beetles [47] and modulate octopaminergic signaling in neurons of cockroaches [48], suggesting neurological disruption as a potential mode of toxic action. A recent computational study suggested that geraniol may interact with voltage-gated sodium channels (VGSCs) in *Ae. aegypti* by binding to amino-acid residues that have not been implicated in *kdr* resistance [34]. Thus, if geraniol induces toxicity by interacting with VGSCs, then it may bind to VGSCs via a mechanism distinct from pyrethroids. This would be consistent with the relatively modest cross-resistance to geraniol detected in larvae and adult females, respectively, compared to that for cypermethrin. The same computational study [34] also suggested that geraniol may interact with glutathione *S*-transferases (GSTs) and α-esterases in *Ae. aegypti*, which have both been implicated in metabolic resistance of mosquitoes to pesticides [49]. Thus, if geraniol indeed interacts with and is a substrate of these detoxification enzymes, then life stage-specific differences in their expression, as occurs in some mosquito species [43,50], could potentially lead to stage-specific differences in detoxification capacities for geraniol. This could potentially explain the different toxic resistance ratios in larvae vs. adult females. Future studies examining the impacts of synergists that inhibit CYP450s (e.g., piperonyl butoxide), GSTs (e.g., diethyl maleate), or esterases (e.g., tribufos) on the toxicity of geraniol may help further elucidate the mechanisms of cross-resistance in larvae of the PR strain.

The present study also demonstrated that geraniol elicited dose-dependent spatial repellency against both PS and PR *Ae. aegypti*. These results were consistent with previous studies that found geraniol repelled *Ae. aegypti* in both arm-in-cage and diffuser-based field assays [15,25]. However, our results conflict with a previous study in *Ae. aegypti* that found geraniol to be ineffective as a repellent when applied to ponies [33].

Notably, adult females of the PR strain possessed modest cross-resistance (2.6 resistance ratio) to the repellency of geraniol, but this was much lower than that for pyrethrum extract (>132 resistance ratio). It should be noted that in the PR strain, a few mosquitoes were knocked down in several replicates of the two highest doses (see Section 2.5), suggesting some degree of vapor toxicity was occurring at these doses. These knocked down mosquitoes were excluded from our repellency calculations. Nevertheless, our repellency findings with geraniol were consistent with a previous study in the same PR strain of *Ae. aegypti* that found various degrees of repellency resistance to pyrethrum extract and pyrethroids (transfluthrin and metofluthrin), as well as other compounds, such as DEET, 2-undecanone, and IR3535, leading the investigators to propose that the pyrethroid resistance mechanisms in the PR strain offer broad resistance to a wide diversity of repellents [43]. It is also important to note that the results for the PR strain of *Ae. aegypti* might not apply to pyrethroid-resistant strains of other mosquito species, because a pyrethroid-resistant strain of *An. gambiae* did not exhibit cross-resistance to geraniol, whereas a carbamate-resistant strain of *An. gambiae* showed strong cross-resistance to geraniol [4]. Thus, the efficacies of various repellents against different insecticide-resistant mosquito species should be confirmed on a species by species (and perhaps a strain by strain) basis.

Presumably, the strong repellency resistance to pyrethrum extract in the PR strain is due to *kdr* mutations that would weaken the interactions between natural pyrethrins and VGSCs; pyrethrins have been shown to drive the majority of the repellent activity associated with pyrethrum extract [51]. As mentioned above, although geraniol was predicted to interact with VGSCs in silico, its binding mechanism is hypothesized to be distinct from that of pyrethroids or pyrethrins [34]. Thus, it is more likely that the modest repellency cross-resistance to geraniol in the PR strain is associated with the metabolic resistance mechanisms. This might also explain the broad repellent cross-resistance to DEET, 2-undecanone, and IR3535, which are not known to modulate VGSCs. However, we cannot rule out that the PR strain also possesses cuticular resistance, which could potentially contribute to the broad repellency cross-resistance in this strain [52]. Future studies examining the impacts of synergists, such as piperonyl butoxide or diethyl maleate, on the repellency of geraniol and other non-pyrethrin/pyrethroid compounds might help further elucidate the mechanisms of broad repellency cross-resistance in adult females of pyrethroid-resistant mosquitoes. Nevertheless, given the relatively weak repellency cross-resistance to geraniol in the PR strain compared to that for pyrethrum extract found in the present study, our data suggest that geraniol substantially overcomes most of the pyrethroid-resistance mechanisms in *Ae. aegypti*. Thus, geraniol has strong potential for use as a repellent against pyrethroid-resistant populations of *Ae. aegypti*.

In conclusion, the results from our study support the notion that geraniol is a potentially valuable natural product for developing novel control tools to combat pyrethroid-resistant mosquitoes. However, it should be noted that the present study evaluated only one representative pyrethroid-susceptible and pyrethroid-resistant strain of *Ae. aegypti*. Thus, future studies in other insecticide-resistant strains and species of mosquitoes are needed to confirm the broad applicability of our findings. The promising results of the present study should also motivate future studies to better characterize the biochemical and physiological modes of action of geraniol as a toxicant and repellent. Furthermore, future studies incorporating synergists (e.g., piperonyl butoxide, diethyl maleate, tribufos) could help further elucidate the mechanisms of modest cross-resistance in the PR strain to the larvicidal and repellent activities of geraniol observed in the present study.

## Data Availability

The raw data supporting the conclusions of this article will be made available by the authors on request.

## Supplementary Materials

The following supporting information can be downloaded at: https://www.mdpi.com/article/10.3390/insects17040385/s1, Figure S1: Double solvent controls (100% acetone on each side) for MART assays. For geraniol experiments, values represent mean ± SEM based on 12 replicates per dose for PS and 6 replicates per dose for PR. For pyrethrum extract experiments, values represent mean ± SEM based on 5 replicates per dose for PS and 8 replicates per dose for PR. Each replicate consisted of one tube of 12 mosquitoes. None of the means were significantly different from zero as determined by a one-sample *t*-test (if data were normally distributed) or Wilcoxon signed-rank test (if data were not normally distributed). The results indicate that in the absence of geraniol or pyrethrum extract, mosquitoes from both strains show no preference for a side of the tube.
