# Supplementary material for: Impact of Pyrethroid Resistance on the Intrinsic Insecticidal Activities of Geraniol Against the Yellow Fever Mosquito, Aedes aegypti
Source: Insects. 2026 Apr 2;17(4):385. doi: 10.3390/insects17040385 (PMC13115756; doi:10.3390/insects17040385)
Supplement: Supplementary file 1 [file insects-17-00385-s001.zip › insects-4190980-supplementary.pdf]

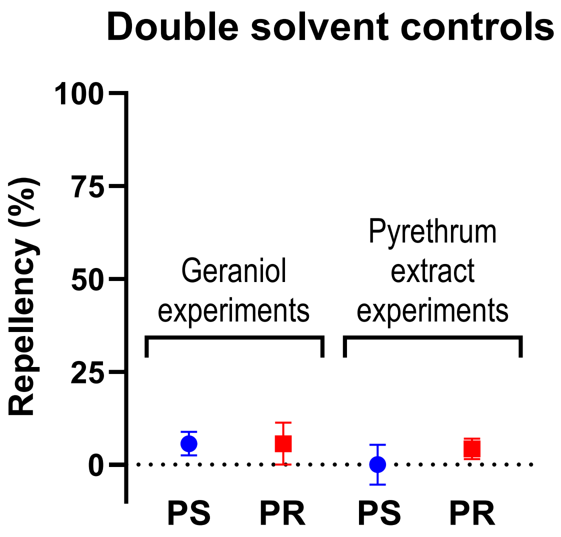

Figure S1. Double solvent controls (100% acetone on each side) for MART assays. For geraniol experiments, values represent mean ± SEM based on 12 replicates per dose for PS and 6 replicates per dose for PR. For pyrethrum extract experiments, values represent mean ± SEM based on 5 replicates per dose for PS and 8 replicates per dose for PR. Each replicate consisted of one tube of 12 mosquitoes. None of the means were significantly different from zero as determined by a one-sample *t*-test (if data were normally distributed) or Wilcoxon signed-rank test (if data were not normally distributed). The results indicate that in the absence of geraniol or pyrethrum extract, mosquitoes from both strains show no preference for a side of the tube.
